# Oral health-related quality of life, sense of coherence and dental anxiety: An epidemiological cross-sectional study of middle-aged women

**DOI:** 10.1186/1472-6831-12-14

**Published:** 2012-06-18

**Authors:** Ulla Wide Boman, Anette Wennström, Ulrika Stenman, Magnus Hakeberg

**Affiliations:** 1 Department of Behavioral and Community Dentistry, Institute of Odontology, The Sahlgrenska Academy, University of Gothenburg, Göteborg, Sweden

**Keywords:** Dental anxiety, Epidemiology, Oral health-related quality of life, Sense of coherence, Women

## Abstract

**Background:**

Few publications report on the relationship between salutogenesis, as measured by the concept of sense of coherence, and oral health-related quality of life. Even less information is to be found when the behavioural aspect of dental anxiety is added. The aim of the present study was to evaluate how oral health-related quality of life is related to sense of coherence and dental anxiety.

**Method:**

The study had a cross-sectional design and included 500 randomly selected women in Gothenburg, Sweden, 38 and 50 years of age, from health examinations in 2004–05. The survey included questionnaires covering global questions concerning socio-economic status, oral health/function and dental care behaviour, and tests of oral health-related quality of life, sense of coherence, and dental anxiety.

**Results:**

High dental anxiety and low sense of coherence predicted low oral health-related quality of life. In addition, socioeconomic status as measured by income, perceived oral functional status as captured by chewing ability and self-reported susceptibility to periodontal disease were also important predictors of oral health-related quality of life.

**Conclusion:**

Dental anxiety and sense of coherence had an inverse relationship with regard to oral health-related quality of life. These associations were stronger than other risk factors for low oral health-related quality of life.

## Background

The concept of salutogenesis, as measured by sense of coherence (SOC) was theorized by Antonovsky and in his book, *Unraveling the mystery of health. How people manage stress and stay well*, published 1987 [1], he discussed the issue of SOC specifically. The core concept of salutogenesis and SOC is to explain why some individuals stay healthy, especially after experiencing highly and long-lasting stressful life situations, while others experience disease and illness. During the past two decades, a considerable number of scientific publications have targeted this concept to reveal possible associations between SOC and different aspects of health and disease [2]. The main findings conclude that there are clear and significant correlations between SOC and several psychological measures, such as depression and anxiety, lifestyle behaviour (*e.g.*, physical activity, dietary habits), and self-perceived health [2]. However, less strong associations are seen between SOC and physical health measures; *i.e.*, objective health or disease markers [2].

These findings are also valid for SOC and oral health status [3-5]. Publications have reported significant correlations between SOC and dental care behaviour and perceived oral health [4,6], albeit less so with regard to the patients’ clinical status. However, a large body of studies is lacking, as most publications emanate from a few population studies from Finland, Sweden and Brazil.

A few studies have reported on the relationship between oral health-related quality of life (OHRQL), SOC and dental anxiety [7]. In a multivariate statistical model, Johansson *et al.*[7] showed how OHRQL was interrelated with dental anxiety and SOC. Both dental anxiety and SOC were significant predictors of OHRQL. In a study from Finland by Savolainen *et al.*[8], SOC was assessed as a determinant for OHRQL, independently of other important risk factors such as objectively measured oral health, oral health behaviour and socio-economic variables.

Dental care or treatment is a special feature inasmuch as anxiety of dental treatment may be an insurmountable factor that may lead to irregular dental attendance behaviour or even avoidance of care, and eventually poor oral health [9,10]. High dental anxiety can thus be inversely related to oral health.

The aim of the present study was to evaluate how oral health-related quality of life is related to sense of coherence and dental anxiety.

## Methods

The Population Study of Women in Gothenburg, Sweden, was initiated in 1968–69. It was a combined medical and dental examination. At the start of the study, the women were 38, 46, 50, 54 and 60 years of age. Subsequent surveys were made in 1980–81, 1992–93 and 2004–05, when new groups of women were invited with the same inclusion criteria as in the previous examinations to ensure representativeness. Detailed information on the sampling procedure has been published previously [11-14], however, a systematic random sampling method was used with women being invited born on day 6, 12, 18 and 24. This study, with a cross-sectional design, included women in Gothenburg, aged 38 and 50 years old, from the survey in 2004–05 where data on SOC and OHIP is available. There were 500 participants (N = 207 and N = 293 38-year-olds and 50-year-olds, respectively) and 346 non-participants. The non-participants in 2004–05 had a lower income and were more often immigrants compared with the participants [14].

Written informed consent was obtained from all participants. The study was approved by the Regional Ethical Review Board at the University of Gothenburg (Dnr 134–05).

The survey included a medical and a dental examination, and self-rated questionnaires including global questions concerning socio-economic status, oral health/function and dental care behaviour, and tests of oral health-related quality of life, sense of coherence and dental anxiety.

Sense of coherence was measured with the short version of the SOC questionnaire which consists of 13 items related to the three interrelated components of SOC; comprehensibility (five items), manageability (four items), and meaningfulness (four items) [1,15]. Each item was scored on a scale from 1–7 points, giving a total range from 13 to 91 points for the SOC score. A higher score indicates a stronger sense of coherence.

Oral health-related quality of life was measured with the Swedish version [16] of the Oral Health Impact Profile (OHIP-14) which consists of 14 items describing several dimensions of health-related quality of life in an oral health context [17]. Each item was scored on a five-degree scale, from 1 = never to 5 = very often, indicating the degree or severity to which individuals perceive their oral conditions/symptoms and effects on life situations. The sum of scores ranges from 14 to 70. The frequency of scores from 1 to 2 (never to seldom) and 3 to 5 (sometimes to very often) were categorized per item into dichotomous variables (0 or 1) and then summed up for all 14 items, giving scores between 0 and 14, with 62.5% of the individuals scoring 0; *i.e.*, having experienced no symptoms or dysfunction at all from their mouth/teeth. A decision was made to dichotomize at a cut-off level of two points; thus, individuals having a score of three or more for this new variable were considered as having problems. This OHIP-14 score was used as the dependent variable in the subsequent statistical analysis. This method of calculating the OHIP-14 score is similar to a method used previously by Savolainen *et al.*[8].

Dental anxiety was measured using the Dental Fear Survey (DFS), which consists of 20 items covering anticipatory anxiety, physiological reactions and situational anxiety [18]. Responses are scored from 1 (no anxiety) to 5 (high intensity of anxiety), giving a total score from 20 to 100. A DFS score of 60 or higher denotes dental anxiety [19], and was used as the cut-off point in this study to detect dental anxiety.

Self-reported oral health was measured with a question where the participants rated their oral health as poor, moderate, good or very good. For the analysis, this variable was dichotomized into poor (poor and moderate) and good (good and very good) oral health. Also included were questions regarding self-reported oral hygiene, chewing ability, self-reported mouth dryness, esthetic aspects of oral status, self-reported susceptibility to caries and periodontitis, and dental visiting habits. These variables were measured on a 4- or 5-degree scale from low to high, but dichotomized (Table 1). The question of regularity of dental care was dichotomized into regular (dental care at least every second year) and irregular (less often).

**Table 1:** Descriptive statistics (proportion %) of self-reported oral health and dental visiting habits with regard to low and high oral health-related quality of life (OHRQL)

**Variables**	**Total group**	**Low OHRQL**	**High OHRQL**	**p**
**N**	493	86	407	
**Self-reported oral hygiene** %				
Poor	10.5	21.2	7.7	0.001
Good	89.5	78.8	92.3	
**Chewing ability** %				<0.001
Poor	14.3	49.4	6.9	
Good	85.6	50.6	93.1	
**Self-reported mouth dryness** %				0.003
Yes	14.0	24.7	11.4	
No	86.0	75.3	88.6	
**Esthetic aspects of oral status** %				<0.001
Poor	17.2	38.8	12.2	
Good	82.8	61.2	87.8	
**Self-reported susceptibility to caries** %				<0.001
Yes	45.9	67.5	39.1	
No	56.1	32.5	60.9	
**Self-reported susceptibility to periodontitis** %				<0.001
Yes	18.1	42.9	12.4	
No	81.9	57.1	87.6	
**Self-reported oral health** %				<0.001
Poor	24.0	58.3	16.2	
Good	76.0	41.7	83.8	
**Dental visiting habits** %				<0.001
Irregular	10.5	32.9	5.7	
Regular	89.5	67.1	94.3	

Marital status was given as not living together (living alone, unmarried, divorced, widowed or married but not living together), or living together (co-habiting, married or in partnership).

Social group was divided into three categories, based on the women's own occupation. This information was transformed according to Carlson’s standard occupation grouping system [20]: low social group (skilled and unskilled workers), medium social group (small-scale employers, lower rank officials, foremen) and high social group (large-scale employers and high or intermediate rank officials).

Educational levels were based on years of school attendance and reported as: low (1–9 years), medium (10–12 years), and high level of education (≥13 years).

Income was measured in thousands of Swedish kronor (SEK). It was then divided into 3 categories; low, medium and high, where low income corresponded to the lowest 20% and high income to the highest 20%.

The statistical analysis consisted of descriptive statistics and inference testing using the *t*-test, the chi-square test, Fisher’s exact test, one-way analysis of variance and multiple logistic regressions using SPSS 19.0. A hierarchical regression modelling strategy was applied by first including socio-economic status (SES) variables, then checking how much variability was accounted for by dental anxiety and SOC, and at the last step, SES, dental anxiety, SOC, and self-reported oral health were included for the full model thereby examining the contribution of each specific measuring area of interest. The test statistic Nagelkerke was used to assess the model fit. The chosen level of significance was *p* < 0.05. The number of individuals included in the analyses varied from 488 to 493 due to some missing answers in the questionnaires.

## Results

Table 2 shows descriptive results concerning SES in relation to OHRQL. The OHRQL was significantly related with low social group, education and income, respectively. Additional analyses concerning the variable age was performed. Thus, significant differences were found between the age groups for education (χ = 12.3, *p* = 0.002), self-reported oral health (Fisher's exact test *p* = 0.018), dry mouth problems (Fisher's exact test *p* = 0.034), susceptibility to periodontitis (Fisher's exact test *p* = 0.006), respectively (data not shown). Thus, the 50-year-old women reported a lower educational level, more dry mouth problems, more susceptibility to periodontitis than the 38-year-old women. Table 3 shows descriptive results concerning OHRQL, SOC and dental anxiety for the total group, on these variables there were no differences between the 38-year old women and the 50-year old women (data not shown).

**Table 2:** Descriptive statistics (proportion %) of socio-economic status with regard to low and high oral health-related quality of life (OHRQL)

**Variables**	**Total group**	**Low OHRQL**	**High OHRQL**	**p**
N	493	86	407	
**Marital status** %				n.s.
Single	50.5	56.5	49.0	
Cohabiting	49.5	43.5	51.0	
**Social group** %				0.006
Low	28.9	41.7	25.7	
Medium	49.7	45.2	50.7	
High	21.4	13.1	23.5	
**Education** %				0.002
Low	8.1	15.3	6.7	
Medium	38.9	47.1	37.4	
High	52.9	37.6	55.9	
**Income** %				<0.001
Low	22.2	34.5	19.2	
Medium	58.2	60.7	58.2	
High	19.6	4.8	22.6	

**Table 3:** Descriptive statistics (mean and standard deviation or proportion %) of oral health-related quality of life (OHRQL measured with OHIP-14), sense of coherence (SOC), dental anxiety (DFS)

**Variables**	**Total**
N	493
**OHRQL**	18.6 (6.9)
high %	82.6
low %	17.4
**SOC**	70.9 (12.4)
**Dental anxiety**	36.2 (16.1)
low %	89.5
high %	10.5

Bivariate analysis applying the dichotomized dependent variable OHRQL (OHIP-14) with the independent variables, showed statistically significant results for all variables except for marital status and age. Sense of coherence showed clear and significant differences with regard to OHRQL, low *vs.* high with mean SOC values of 62.5 SD = 13.8 and 72.7 SD = 11.2 (t = 7.3, *p* < 0.001), respectively; dental anxiety, low *vs.* high with mean DFS values of 71.7 SD = 12.1 and 64.3 SD = 12.6 (t = 4.2, *p* < 0.001). Moreover, low *vs.* high OHRQL was significantly correlated with dental anxiety using the continuous scale. Mean DFS values were 49.1 SD = 22.0 and 33.4 SD = 13.1 (t = 6.3, *p* < 0.001 under the assumption of no equal variances), respectively.

Also, problems with oral hygiene, with chewing ability, with dry mouth, with esthetic aspects of oral status, high self-reported susceptibility to caries and to periodontitis, poor self-reported oral health, and irregular dental visiting habits were all associated with poor OHRQL (Table 1).

Three different multivariate models of logistic regression are displayed in Table 4. Model 1 tested the influence of the SES variables on the dependent variable of OHRQL. The strongest factor was income with high ORs and a gradient effect; the lower the income, the higher the risk of poor OHRQL. For the subsequent models only income was included concerning SES. Model 2 revealed the association between the behavioural factors of dental anxiety, dental visiting habits and sense of coherence. Income as a confounder was still a significant predictor of OHRQL. High dental anxiety and irregular dental care had large ORs, indicating an almost 4 times higher risk of poor OHRQL in comparison with lower levels of these behavioural factors. Higher SOC scores indicated a protective effect against poor OHRQL. The last analysis, according to model 3, included some significant factors from the bivariate analysis, such as oral function measured by chewing ability and self-reported susceptibility to caries and periodontitis, to elucidate the degree of proneness to disease development. Other variables that were statistically significant for the outcome in model 3 were dental anxiety and SOC. The explanatory power of the models was indicated by the Nagelkerke test statistic and revealed an increase from 0.10 to 0.45 from model 1 to model 3, respectively.

**Table 4:** Hierarchical logistic regression models using the oral health-related quality of life measure OHIP-14 as the dependent variable with SES, SOC, dental anxiety, self-reported oral health, and dental visiting habits as independent variables. Odds ratios and 95% confidence intervals

	**Model 1**	**Model 2**	**Model 3**
**Nagelkerke**	0.10	0.28	0.45
**Age** (50)	1.2 (0.8-2.1)		
**Marital status,** single	1.5 (0.9-2.4)		
**Social group**			
Medium	1.2 (0.6-2.5)		
Low	1.4 (0.6-3.3)		
**Education**			
Medium	1.3 (0.7-2.3)		
Low	2.0 (0.9-4.7)		
**Income**			
Medium	4.1 (1.4-12.1)	3.6 (1.2-10.6)	2.1 (0.6-7.4)
Low	6.5 (2.1-20.1)	4.1 (1.3-12.8)	3.0 (0.9-9.1)
**Dental anxiety,** high		3.7 (1.8-7.4)	3.5 (1.6-8.0)
**Dental visits,** irregular		3.9 (1.9-7.8)	1.6 (0.7-3.8)
**SOC**		0.95 (0.94-0.97)	0.96 (0.93-0.98)
**Chewing ability,** poor			5.1 (2.4-10.8)
**Self-reported oral health,** poor			2.2 (1.1-4.5)
**Self-reported susceptibility to caries,** yes			1.7 (0.9-3.2)
**Self-reported susceptibility to periodontitis,** yes			2.4 (1.2-4.9)

## Discussion

Very few publications in the scientific literature report on the relationship between salutogenesis, as measured by the concept of sense of coherence, and oral health-related quality of life. Even less information is to be found when the behavioural aspect of dental anxiety is added to the previous two concepts [7]. Thus, the aim of the present epidemiological study was to evaluate how OHRQL; *i.e.*, the perceived well-being of individuals relative to their oral health, was correlated with the sense of coherence and dental anxiety. The notion behind such a relationship was that SOC would be positively associated and dental anxiety negatively correlated with OHRQL. The results from both the bivariate and multivariate statistical modeling confirmed these assumptions.

There is a lack of information regarding the relationship between health-related quality of life and SOC through population-designed epidemiological studies. The majority of analyses concern health-related quality of life and SOC for specific diseases, such as rheumatic disorders, heart disease and mental illness [2]. However, most studies report that poor health-related quality of life is associated with lower SOC values [2]. The correlation between SOC and OHRQL has been described, as far as the authors know, in only two reports: one from Finland [8] and one from Sweden [7], both regarding adult individuals. Both studies show significant relationships between SOC and OHRQL in multivariate models adjusted for common risk factors and confounders such as age, gender, socio-economic status, dental care behaviour and oral status. However, only Johansson *et al.*[7] also included dental anxiety in the models and that variable was, in fact, stronger than SOC. Dental anxiety and SOC were inversely related to OHRQL; higher SOC scores predicted better OHRQL, while higher dental anxiety indicated lower levels of OHRQL. The present study revealed results similar to those of Johansson *et al.*[7] and Savolainen *et al.*[8]. In addition, our model 3 was supplemented with self-reported measures of oral health factors that revealed high and significant odds ratios. Moreover, the difference in ORs with regard to SOC in models 2 and 3 was marginal, implying that the effect of SOC on OHRQL was independent of other potential explanatory factors. This fact may point towards SOC having a direct path to the individual’s self-perceived well-being and/or health and one may speculated if SOC could act as a clinical measure predicting oral health.

Sense of coherence as a concept has been studied with regard to other psychological concepts. Depression and general anxiety have been found to be strongly associated with SOC [21,22]. Some researchers argue that SOC mirrors these common psychological conditions to some degree and question the concept of salutogenesis as such. Depression and anxiety are well-known risk factors for a spectrum of diseases and conditions [23,24].

Limitations of the study were that only females were included, the cross-sectional survey design and the narrow age range. However, positive characteristics of the study were the random selection of women, acceptable measures of OHRQL, dental anxiety and SOC, and a moderate non-participation rate. The non-participants had lower income and were more often immigrants than the participants. It may be speculated that the results have underestimated the effects of dental anxiety and sense of coherence, as other studies have shown higher dental anxiety and lower sense of coherence among these subgroups [25,26].

## Conclusions

The conclusion from this study was that high dental anxiety predicted low oral health-related quality of life and that a strong sense of coherence protected against low oral health-related quality of life. Thus, dental anxiety and sense of coherence had an inverse relationship with regard to oral health-related quality of life. In addition, socioeconomic status as measured by income, perceived functional status as captured by chewing ability and self-reported susceptibility to periodontal disease were also important predictors of oral health-related quality of life.

## Competing interests

The authors declare that they have no competing interests.

## Authors’ contributions

UWB performed the analysis, the interpretation of results and wrote the manuscript. AW planned and carried through the examinations together with US. AW also participated in the analysis. MH planned the dental study, participated in the data collection, analysis and the draft of the manuscript. All authors have read and approved of the final manuscript.

## Pre-publication history

The pre-publication history for this paper can be accessed here:

http://www.biomedcentral.com/1472-6831/12/14/prepub
